# NMR metabolome of *Borrelia burgdorferi in vitro* and *in vivo* in mice

**DOI:** 10.1038/s41598-019-44540-5

**Published:** 2019-05-29

**Authors:** Otto Glader, Elina Puljula, Johanna Jokioja, Maarit Karonen, Jari Sinkkonen, Jukka Hytönen

**Affiliations:** 1 Institute of Biomedicine, University of Turku, Turku, Finland; 2 Instrument Centre, Department of Chemistry, University of Turku, Turku, Finland; 3 Natural Compound Chemistry, Department of Chemistry, University of Turku, Turku, Finland; 4 Clinical Microbiology, Turku University Hospital, Turku, Finland

**Keywords:** Infectious-disease diagnostics, Bacterial host response

## Abstract

Lyme borreliosis (LB), caused by bacteria of the *Borrelia burgdorferi* sensu lato (*Borrelia)* species, is the most common tick-borne infection in the northern hemisphere. LB diagnostics is based on clinical evaluation of the patient and on laboratory testing, where the main method is the detection of *Borrelia* specific antibodies in patient samples. There are, however, shortcomings in the current serology based LB diagnostics, especially its inability to differentiate ongoing infection from a previously treated one. Identification of specific biomarkers of diseases is a growing application of metabolomics. One of the main methods of metabolomics is nuclear magnetic resonance (NMR) spectroscopy. In the present study, our aim was to analyze whether *Borrelia* growth *in vitro* and infection *in vivo* in mice causes specific metabolite differences, and whether NMR can be used to detect them. For this purpose, we performed NMR analyses of *in vitro* culture medium samples, and of serum and urine samples of *Borrelia* infected and control mice. The results show, that there were significant differences in the concentrations of several amino acids, energy metabolites and aromatic compounds between *Borrelia* culture and control media, and between infected and control mouse serum and urine samples. For example, the concentration of L-phenylalanine increases in the *Borrelia* growth medium and in serum of infected mice, whereas the concentrations of allantoin and trigonelline decrease in the urine of infected mice. Therefore, we conclude that *Borrelia* infection causes measurable metabolome differences *in vitro* and in *Borrelia* infected mouse serum and urine samples, and that these can be detected with NMR.

## Introduction

Lyme borreliosis (LB) is a multi-organ infectious disease caused by spirochetes of *Borrelia burgdorferi* sensu lato species (later *Borrelia*). *Borrelia* is a common tick-borne pathogen in the northern hemisphere, making LB the most important tick-borne infectious disease in this area^1^. In the USA, the estimated number of new LB cases is 300,000 annually. In Europe, LB incidence is the highest in Scandinavia and central Europe. For example, in Finland, the annual incidence of disseminated LB is approximately 2,300 cases, and the number of local skin infections is three to five times that of the disseminated infections^2^.

The clinical manifestations of LB can be classified as the local skin infection at the tick bite site (erythema migrans; EM), and as various forms of the disseminated infection, namely infection of the nervous system (Lyme neuroborreliosis), infection of the joints (Lyme arthritis), chronic skin disorders and carditis. The diagnosis of a case of disseminated LB is established based on clinical manifestations and laboratory tests^1^. The mainstay of LB laboratory testing is the detection of *Borrelia*-specific antibodies in patient serum. This method performs well in the first episode of disseminated LB in a patient. However, antibodies in patient serum remain elevated after treatment for up to several years, leading to substantial seropositivity in the population (e.g. 3.9% in Finland)^3^. Therefore, an obvious shortcoming in LB serodiagnostics is its inability to differentiate an ongoing case of LB from a previously treated one^4,5^.

Metabolomics is one approach in the field of the so-called omics sciences. It uses cutting-edge analytical chemistry techniques combined with computational methods to characterize complex mixtures of molecules. One application of metabolomics is the global analysis of low molecular mass biological molecules in order to identify biomarkers of specific disease states^6,7^. Using metabolomics-based approaches, biomarker panels have been identified in a variety of human conditions: for example, in coronary heart disease, diabetes, nephropathy and in various cancers^8–10^. Also, in infections such as tuberculosis, typhoid fever, dengue virus infection^11–13^, malaria and schistosomiasis^14^, metabolomics has been utilized for biomarker profile discovery. Importantly, there are three previous publications with promising results of LB metabolomics of human samples^15–17^.

The main methods of metabolomics are gas chromatography-mass spectrometry, liquid chromatography-mass spectrometry, and nuclear magnetic resonance spectroscopy (NMR). NMR is an analytical method based on the magnetic properties of atom nuclei. NMR active nuclei (e.g. ^1^H, ^13^C, ^15^N, and ^31^P) are commonly found in biological samples which makes the methodology highly usable in biomarker research^6^. NMR has several advantages: the sample preparation is relatively easy and not time-consuming, and during NMR measurements the samples are not destroyed, which allows multiple measurements of one sample. Probably the most important advantage of NMR is the possibility to recognize metabolites based on their chemical properties^6^. NMR has been shown to be an extremely useful tool in metabolomics, as different metabolites can be identified and quantified even from complex mixtures^18^.

The present study was initiated to evaluate whether NMR can be used to detect *Borrelia-* specific metabolites *in vitro* and *in vivo* in mice. Our results show that, indeed, *Borrelia* growth causes NMR-demonstrable metabolome changes in the *in vitro* culture medium samples, and importantly, also in *Borrelia*-infected mouse serum and urine samples.

## Results

**Figure 1:**
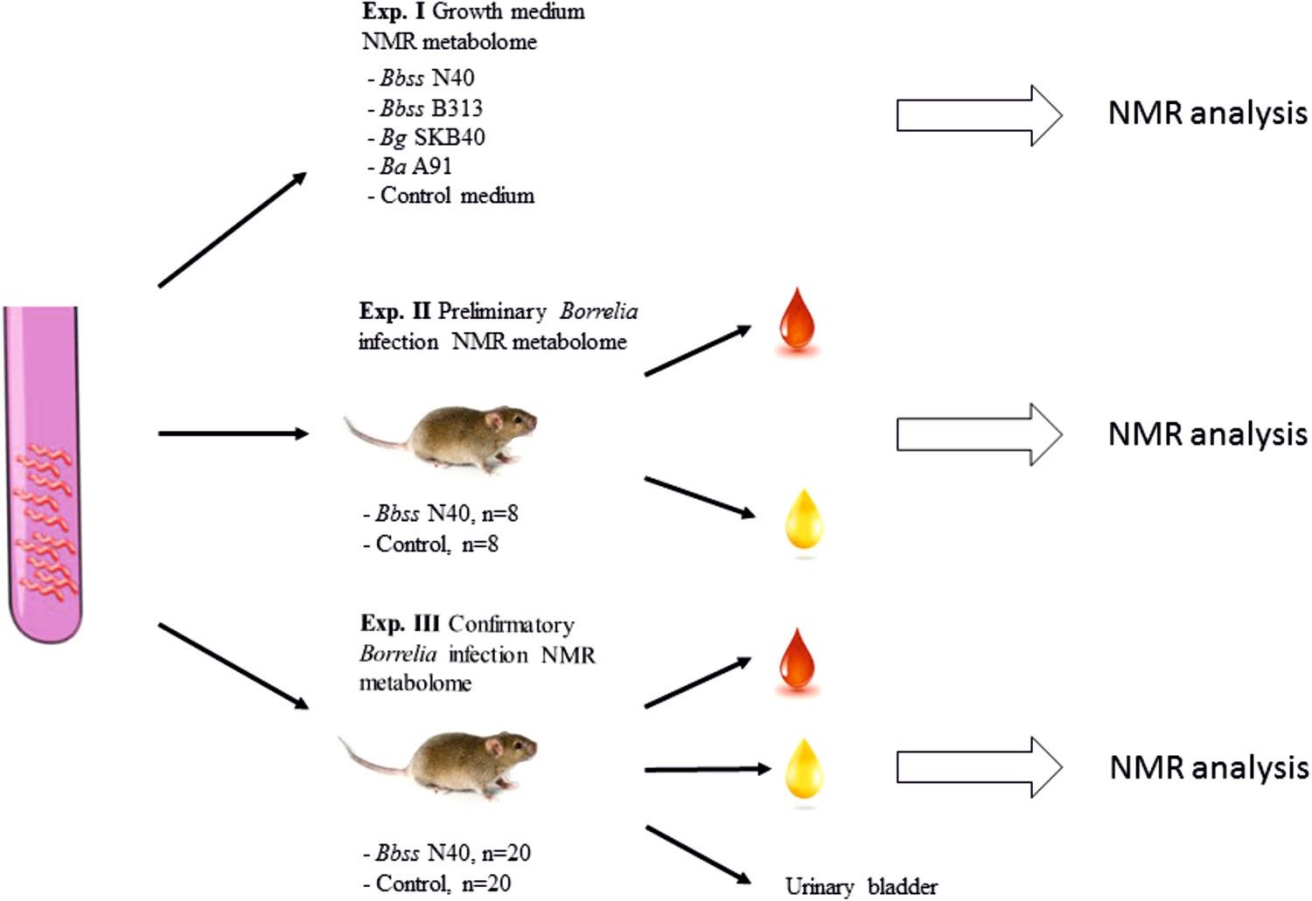
Overall setup of the study. The study consisted of three experiments. In Experiment I, the *in vitro* growth medium metabolomes of different *Borrelia* strains (*Bbss* N40, *Bbss* 313, *Bg* SKB40 and *Ba* A91) were analyzed using NMR. In Experiment II, *in vitro* grown *Bbss* N40 were used to infect eight mice, and eight mice were used as uninfected controls. After 4 weeks follow up, the mice were sacrificed and serum samples were collected for NMR analysis. Urine samples were collected on three consecutive days before killing on the week 4. Ear skin samples were collected for *Borrelia* culture. In Experiment III, *in vitro* grown *Bbss* N40 were used to infect 20 mice, and 20 mice were used as uninfected controls. After 4 weeks follow up, the mice were sacrificed, and serum samples and urinary bladders were collected for NMR analysis. Urine and ear skin samples were collected as in Experiment II.

This study included three separate experiments (Fig. 1). The study was initiated with an *in vitro* experiment (Exp. I). Following the promising results of Experiment I, two sets of animal experiments (Exp. II and Exp. III) were performed, resulting in the *in vivo* NMR metabolome of mouse *Borrelia* infection.

### Experiment I: *In vitro* NMR metabolome of *Borrelia*

In Experiment I, *in vitro* growth medium NMR metabolomes, (measured by Carr-Purcell-Meiboom-Gill pulse sequence (CPMG); see Materials and methods) of different *Borrelia* strains (*B. burgdorferi* ss N40 (*Bbss* N40), *B. burgdorferi* ss 313 (*Bbss* 313), *B. garinii* SKB40 (*Bg* SKB40) and *B. afzelii* A91 (*Ba* A91)) were compared. The differences between *Borrelia* growth- and control media were detected by PCA (principal component analysis) and PLS-DA (partial least square means discriminant analysis) and then confirmed by univariate analysis (Fig. S1). The PCA model, of which the first principal component explains 72% of the total variation, separated the samples into five distinguished groups (PCA: *R*^2^ = 0.99, *Q*^2^ = 0.95 and PLS-DA: *R*^2^ = 0.98, *Q*^2^ = 0.97). *R*^2^ represents the variation of the model whereas *Q*^2^ is an estimate of the predictive ability of the model.

**Figure 2:**
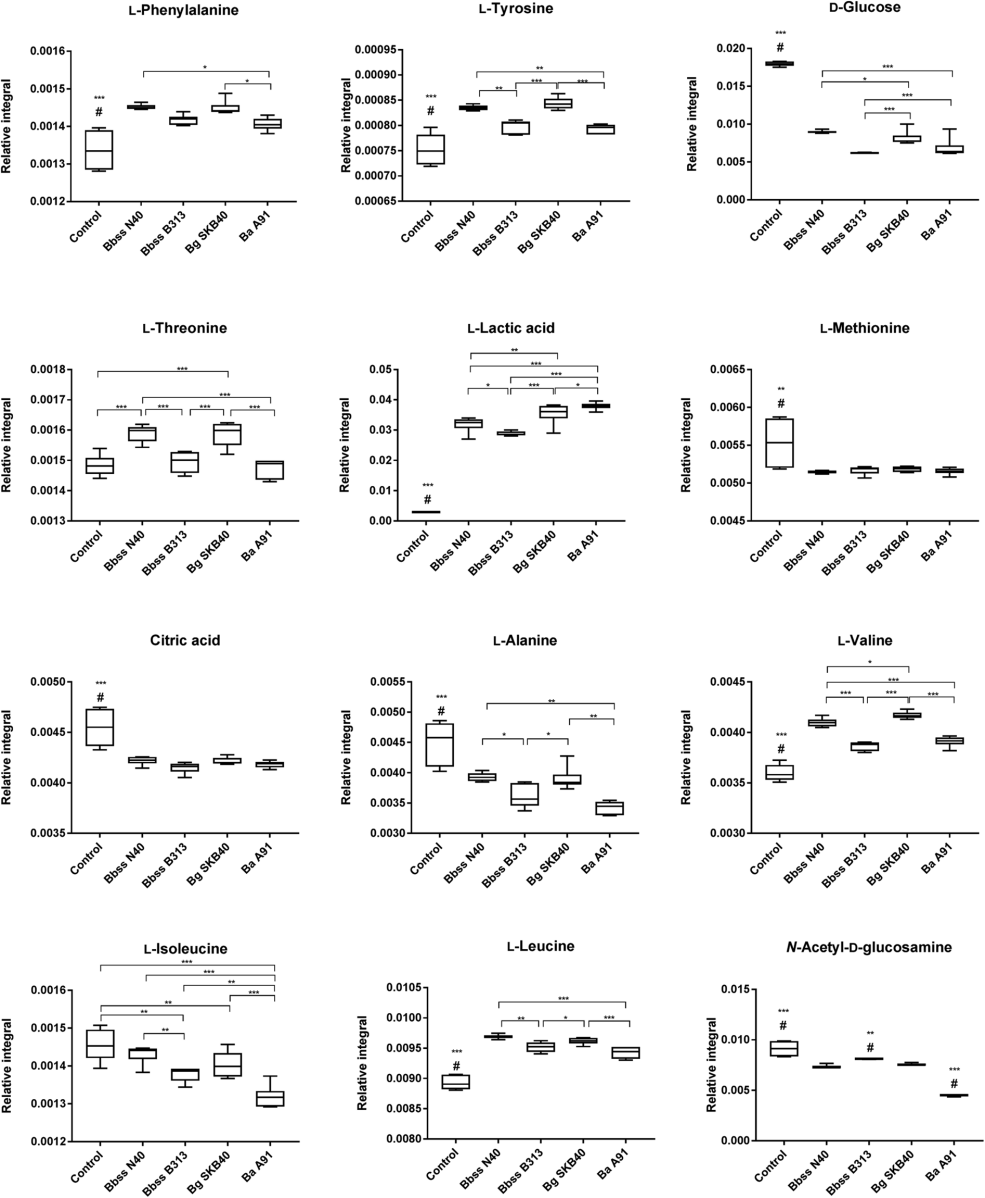
Metabolites in control and *Borrelia* growth medium samples. Boxplot figures present the means of relative integrals of metabolites, standard deviations and 95% confidence intervals. Statistically significant difference between samples is indicated by asterisks (*<0.05, **<0.01, ***<0.001). If a group is statistically significantly different from all other groups it is annotated with #.

Statistically significant differences were observed in the concentrations of several amino acids and energy metabolites (Fig. 2). The results of this experiment revealed that *Borrelia* growth increases the concentrations of L-phenylalanine, L-tyrosine, L-threonine, L-valine, L-leucine and L-lactic, and decrease the concentrations of L-methionine, L-alanine, L-isoleucine, D-glucose, citric acid and N-acetyl-D-glucosamine in the culture media compared to the control medium. This experiment also demonstrated that there were differences between the *Borrelia* genotypes. *Bbss* N40 and *Bg* SKB40 caused a similar change in the concentration of the metabolites forming one pattern, whereas *Bbss* B313 and *Ba* A91 formed their own pattern (Fig. S1). All of these differences were statistically significant at least between control and *Borrelia* incubated medium samples, but in the most of the metabolites also between different *Borrelia* strains (Fig. 2). In total, 23 metabolites were identified in the growth medium samples (Table S1).

### Experiment II: Preliminary metabolome analysis of *Borrelia*-infected and control mice

Because the results of Experiment I demonstrated that NMR can be used to detect *Borrelia* growth-induced metabolome differences *in vitro*, a preliminary mouse study (Exp. II) with eight *Bbss* N40-infected and eight control mice was performed (Fig. 1). The mice were followed up for four weeks, after which the animals were sacrificed and total blood collected. During the last week of the experiment, urine samples were also collected. The urine collection was successful in 10/16 animals. *Borrelia* culture of ear skin samples revealed that all infected mice were *Borrelia* positive, while control animals were culture negative.

**Figure 3:**
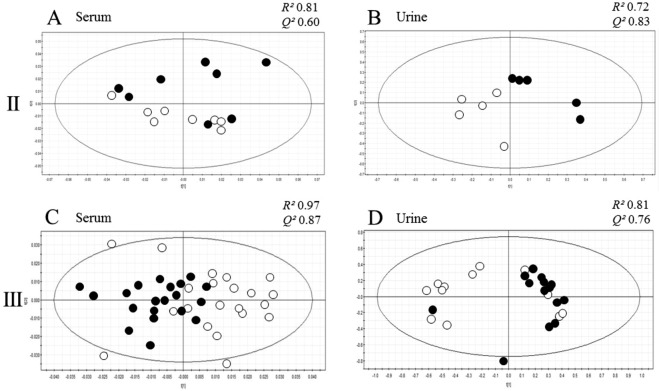
Metabolome differences of *Borrelia* infected and control mice. Scores plot figures of multivariate principal component analyses (PCA) of Experiment II and III serum and urine samples (**A**: Exp. II serum, **B**: Exp. II urine, **C**: Exp. III serum, **D**: Exp. III urine) show the difference between infected mouse (black circles) and control mouse metabolomes (empty circles). *R*^2^ and *Q*^2^ values, representing the reliability of the model of the model, are shown above each scatter plot. The values indicate that the results of all analyses, except the PCA of urine in Experiment II, are reliable.

Based on CPMG spectra of the serum samples, the infected and control groups differed in the metabolite composition in the sera (Fig. 3A, Table 1, Fig. S2A). The differences between *Borrelia* infected and control animal sera were detected by PCA and PLS-DA and then confirmed by univariate analysis In PCA, the *R*^2^ value was 0.81 and the *Q*^2^ 0.60, which means that results are not entirely reliable since the difference between *R*^2^ and *Q*^2^ is slightly over 0.2. In PLS-DA, *R*^2^ value was 0.86 and the *Q*^2^ 0.75. In PCA, the first principal component explained 63% of the total variation. Statistical significance of the metabolite concentration differences observed in the PCA and PLS-DA were determined, and metabolites with statistically significant concentration difference between infected and control mice are presented in Table 1, along with the relative integral means, SD values and fold change values.

**Table 1:** Serum metabolites differing in concentration in the infected and control mice in Experiments II and III based on PCA.

Metabolite	Trend	Statistical significance, p-value	Control serum mean (SD)	Infected serum mean (SD)	Serum fold change	¹H signal ppm
*Amino acids*						
L-phenylalanine	↑	0.06/0.001	0.0110 (0.0066)/0.0971 (0.0329)	0.0159 (0.0094)/0.1402 (0.0066)	1.45/1.44	**7.435**
L-threonine	↑	0.0004	0.0018 (0.0003)	0.0022 (0.0005)	1.27	**4.335**
**L-glutamine**	↓	0.049/0.02	0.0065 (0.0008)/0.0129 (0.0029)	0.0054 (0.0012)/0.0109 (0.0020)	1.20/1.18	**2.025**
L-leucine	↑	0.01	0.0071 (0.0008)	0.0097 (0.0006)	1.36	**0.965**
*Energy metabolites*						
**D-glucose**	↑	0.04/0.0001	0.0176 (0.0026)/0.0127 (0.0016)	0.0206 (0.0026)/0.0157 (0.0018)	1.17/1.23	Multiple signals
**L-lactic acid**	↓	0.02/0.006	0.1284 (0.0149)/0.1950 (0.0215)	0.1074 (0.0063)/0.1469 (0.0167)	1.20/1.32	4.125, **1.335**
Citric acid	↑	0.5/0.0001	0.0039 (0.0011)/0.0708 (0.0029)	0.0046 (0.0021)/0.1368 (0.0041)	1.18/1.93	**2.695**
*Others*						
**Urea**	↓	0.04/0.03	0.0082 (0.0020/0.0114 (0.0032)	0.0056 (0.0023)/0.0095 (0.0019)	1.46/1.19	**5.325**
2-hydroxybutyric acid	↓	0.01	0.0290 (0.0059)	0.0245 (0.0044)	1.18	1.735, **1.595**, 0.895

The same differences were observed also using PLS-DA. Metabolites occurring in higher concentration in infected mice are annotated with an upward arrow (↑), whereas metabolites occurring in higher concentration in control mice are annotated with a downward arrow (↓). Metabolites differing statistically significantly in both Experiments II and III are indicated by bold whereas concentration of non-bold metabolites differed only in Experiment III. Statistical significance of metabolite differences is presented as p-values. In addition, relative means of signal integrals, SDs and fold changes are presented. If a metabolite difference is detected in both experiments, p-values, means, SDs and fold changes of both experiments are presented (Exp II/Exp III). Signal positions, by which the metabolite identification was made, are presented as ppm values. Signals based on which the statistical analyses were made are indicated by bold.

Ten urine samples were analyzed using 1D-Nuclear Overhauser Effect Spectroscopy (1D-NOESY) method. Five of the samples were from the infected and five from the control mice. Based on these measurements, the metabolite composition of the urine samples of the infected mice differed from the composition of the control mouse samples as shown in Fig. 3B. In the PCA of urine metabolite data, the *R*^2^ value was 0.72 and the *Q*^2^ value 0.83, and in PLS-DA the *R*^2^ value was 0.74 and the *Q*^2^ value 0.81. The *R*^2^ value, which describes the reliability of the multivariate analysis, is just below the border value of 0.80 in both cases, likely due to the low sample number. In PCA, the first component explained 68% of the total variation. Metabolites with statistically significant concentration difference between infected and control mice are presented in Table 2.

**Table 2:** Urine metabolites differing in concentration in the infected and control mice in Experiments II and III. Metabolites occurring in higher concentration in infected mice are annotated with an upward arrow (↑), whereas metabolites occurring in higher concentration in control mice are annotated with a downward arrow (↓).

Metabolite	Trend	Statistical significance, p-value	Control urine mean (SD)	Infected urine mean (SD)	Urine fold change	¹H signal ppm
*Amino acids*						
L-glutamine	↑	0.08/0.002	0.0054 (0.0004)/0.0018 (0.0006)	0.0059 (0.0003)/0.0025 (0.0003)	1.09/1.39	**2.025**
L-glycine	↓	0.4/0.03	0.0382 (0.0073)/0.0284 (0.0162)	0.0341 (0.0074)/0.0284 (0.0162)	1.12/1.48	**3.275**
*Energy metabolites*						
Acetic acid	↑	0.02/0.25	0.0051 (0.0003)/0.0074 (0.0006)	0.0056 (0.0001)/0.0074 (0.0006)	1.11/1.03	**1.925**
*Aromatic compounds*						
Allantoin	↓	0.8/0.01	0.0282 (0.0048)/0.0119 (0.0074)	0.0277 (0.0028)/0.0119 (0.0074)	1.02/1.66	**5.385**
**Trigonelline**	↓	0.01/0.047	0.0020 (0.0014)/0.0022 (0.0002)	0.0005 (0.0002)/0.0013 (0.0002)	4.00/1.69	**8.125**, 8.084
Hippuric acid	↓	0.2/0.002	0.0074 (0.0021)/0.0031 (0.0016)	0.0059 (0.0008)/0.0031 (0.0016)	1.25/2.03	7.845, **7.555**
Indoxyl sulphate	↓	0.046/0.1	0.0072 (0.0008)/0.0051 (0.0008)	0.0048 (0.0015)/0.0051 (0.0008)	1.50/1.64	**7.195**
*Others*						
Creatinine	↑	0.02/0.5	0.0121 (0.0009)/0.018 (0.0027)	0.0177 (0.0046)/0.018 (0.0027)	1.46/1.13	**3.935**, 3.041

Metabolites differing statistically significantly in both Experiments II and III are indicated by bold whereas concentration of non-bold metabolites differed only in Experiment II or III. Statistical significance of metabolite differences is presented as a p-value. In addition, means of the relative signal integrals, SDs and fold changes of both experiments are presented (Exp II/Exp III). Signal positions, by which the metabolite identification was made, are presented as ppm values. Signals based on which the statistical analyses were made are indicated by bold.

All identified metabolite differences shown in Tables 1 and 2 were quantitative, meaning that no compound was totally lacking (below the detection limit of NMR) in any sample. In addition to the described major differences in Tables 1 and 2, some smaller differences occurred between the infected and control mice, especially in the spectrum area 6.5–9 ppm, but due to the low concentration variability of these molecules, we were not able to determine their identity.

### Experiment III: Confirmatory metabolome analysis of *Borrelia*-infected and control mice

Due to the observed metabolome differences in Experiment II, we conducted a confirmatory mouse experiment as shown in Fig. 1. We also extended our study to semi-solid state NMR analysis of urinary bladders of the mice.

In this experiment, 40 mice in total were used. Half of them were infected and the other half served as control animals. Ear skin sample culture verified that all infected mice were *Borrelia* positive, while control animals were culture negative.

**Figure 4:**
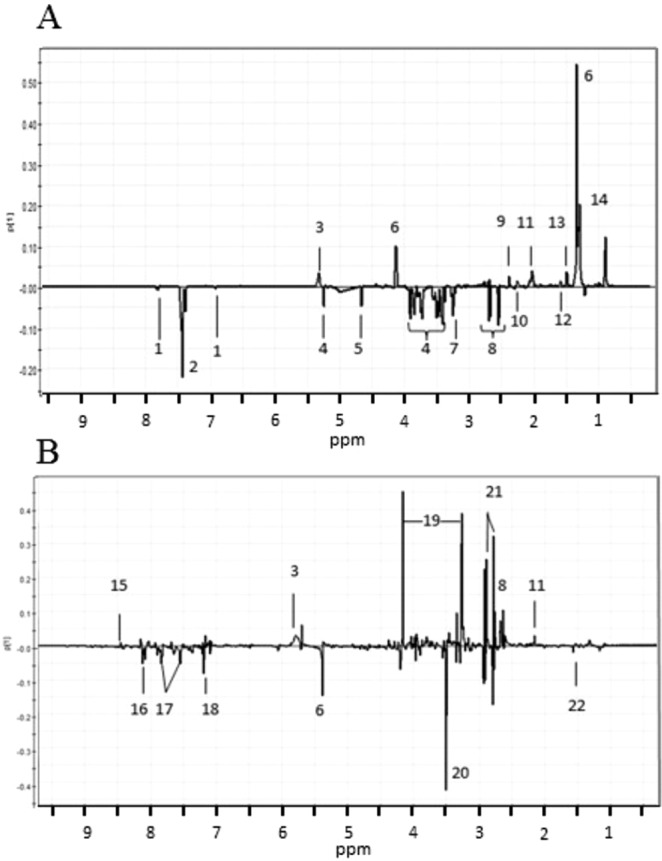
Loadings plots of the serum (**A**) and urine (**B**) analyses of Experiment III show the differentiating metabolites. Positive y-axis represents metabolites occurring in higher concentrations in the control mice, and negative y-axis represents metabolites occurring in higher concentrations in the infected mice. Identified metabolites are: 1: L-histidine, 2: L-phenylalanine, 3: urea, 4: D-glucose, 5: L-threonine, 6: L-lactic acid, 7: carnitine, 8: citric acid, 9: pyruvic acid, 10: L-valine, 11: L-glutamine, 12: 2-hydroxybutyric acid, 13: L-alanine, 14: L-leucine, 15: formic acid, 16: trigonelline, 17: hippuric acid, 18: indoxyl sulphate, 19: allantoin, 20: creatinine, 21: L-glycine, 22: acetic acid.

Also in this experiment, control and infected mice differed based on the metabolite composition of both serum and urine samples as shown in Fig. 3C,D (PCA) and in Fig. S2C and D (PLS-DA). In the PCA of the CPMG analysis of the sera, the *R*^2^ value was 0.97 and *Q*^2^ was 0.87, and in the PLS-DA *R*^2^ value was 0.98 and the *Q*^2^ value 0.89. Thus, the results of Experiment III are in line with the results of Experiment II. In PCA, the first principal component explained 81% of the total variation. Statistical significance of the concentration differences was determined as in Experiment II for both serum and urine metabolites. P-values, means, SDs and fold change values of statistically significantly differing metabolites are presented in Tables 1 and 2. All PCA identified metabolites are shown in Fig. 4. Differences observed in the PLS-DA analysis are presented in Fig. S3. The same metabolite differences were observed using both multivariate analysis methods, and therefore the same annotations are used in Figs 4 and S3.

Major differences between the infected and control serum samples were mainly similar to the differences observed in Experiment II. However, several additional differentiating metabolites were also identified (mainly essential amino acids) due to the larger sample number and refined CPMG analysis protocol.

In this experiment, 27 urine samples were analyzed, 14 of which were from the infected mice and 13 from the control mice. 1D-NOESY measurements of the urine samples clearly showed that the metabolites in the urine of *Borrelia*-infected mice differed from the metabolites in the samples of the control mice (Fig. 3D). The statistically significantly differing metabolites are listed in Table 2 and all metabolites observed are shown in the loadings plots (PCA: Fig. 4B and PLS-DA: Fig. S3B). These results are mainly in line with the urine metabolite results of Experiment II, but due to the bigger number of samples, the results are more reliable. In PCA, the first principal component explained 79% of the total variation.

As a part of Experiment III, the urinary bladders of mice (n = 40) were analyzed with semi-solid state NMR. No considerable differences between the urinary bladders of the infected and the control animals could be detected with PCA (Fig. S4) or with PLS-DA (data not shown).

## Discussion

In this study, we evaluated whether NMR can be used to detect *Borrelia*-specific metabolites *in vitro* and *in vivo* in mice. Our results show that, indeed, *Borrelia* infection causes metabolome differences both in BSK II growth medium incubated with and without bacteria, and in control and *Borrelia*-infected mouse serum and urine samples. On the other hand, no differences between *Borrelia*-infected and control bladder tissues samples were found.

Recently, Molins *et al*. and Pegalajar-Jurado *et al*. have elegantly shown that LB-specific metabolite panels can be identified in human LB patient serum and urine samples using a liquid chromatography-mass spectrometry (LC-MS) approach^15–17^. NMR, on the other hand, has not been previously used in LB metabolomics studies. However, we know that NMR has been successfully applied, for example, in the identification of biomarker profiles for malaria and schistosomiasis^14,19,20^. Especially concerning malaria, NMR has proven to be very useful, since an entirely new malaria-related molecule has been identified^19^. Based on these previous achievements, we decided to conduct our LB *in vitro* and mouse experiments. Importantly, samples originating from laboratory mice have the benefit over human patient samples that the housing conditions and nutrition of the animals are controlled and standardized, the time point of sample collection in regard to the duration of the infection is similar, and the infection status of mice can be confirmed by tissue *Borrelia* culture. There are also some disadvantages in the mouse model. Mice have slightly different metabolic routes than humans, which may cause differences in the metabolomes of *Borrelia* infected mice and humans. Therefore, the metabolite signatures observed in mice may not be present in human patients.

NMR was selected as the method because it provides untargeted data allowing the identification of all molecules of potential interest, and due to the non-destructive nature of the measurements, which is important considering the not-too-large number of mice (in accordance with the 3Rs policy), limited sample volume, and our setup in which the samples were first analyzed multiple times during the method validation.

In general, NMR as a method in metabolomics has its pros and cons^6^. One benefit of the method is the fact mentioned above that using NMR, it is possible to measure one sample multiple times. Measuring the samples is also fast, and less validation, internal standardization and instrument calibration than in LC-MS is required. In addition, different NMR instruments in different laboratories result in robust, identical results. The most important quality of NMR is the reliable recognition of metabolites based on their chemical bond properties. Unfortunately, NMR has also some major drawbacks. The sample volume required is relatively high, which leads to challenges in animal experimentation. Although NMR can recognize most organic molecules, inorganic compounds like salts can’t be identified. The most important disadvantage of NMR is the low sensitivity^6^. In this study we used three different NMR instruments suitable for different sample materials. Our 400 MHz instrument is designed for solid samples, 500 MHz for simple sample materials like the growth medium, and 600 MHz with cryoprobe for complex biological samples like serum and urine^21^.

NMR methodology allows the use of different types of pulse sequences to collect different type of information. In biological samples, like serum and urine, the most problematic signal is the water signal, which covers all other signals and makes the interpretation of the spectrum challenging. Broad signals caused by large molecules, like proteins, are also problematic. These signals are difficult to identify, and they may also hide other signals^21^. Therefore, different pulse sequences have been developed to eliminate the interference of these signals. For example, the Carr-Purcell-Meiboom-Gill (CPMG) pulse sequence is developed to fade the broad signals. The 1D-Nuclear Overhauser Effect Spectroscopy (NOESY) pulse sequence is used for solvent signal suppression in the case when no wide signal suppression is required, such as in urine samples, where no or only a little proteins and lipids are present. Based on these properties of different NMR instruments and pulse sequences, we used the 500 MHz instrument and the CPMG pulse sequence in Experiment I; the 600 MHz instrument with cryoprobe and automatic sample changer for the analysis of serum (CPMG pulse sequence) and urine samples (NOESY pulse sequence) in Experiments II and III; and the 400 MHz instrument with the CPMG pulse sequence for the analysis of the urinary bladder samples in Experiments III.

In Experiment I, statistically significant differences (Fig. 2) in the metabolite profiles of the *in vitro*-grown bacterial strains and control samples were detected. These differences are mainly due to the conversion of glucose in glycolysis of the bacteria into lactic acid, and the lactic acid is then excreted outside of the bacterial cell. However, in addition to energy metabolites, statistically significant differences in the concentrations of several amino acids were also observed. Overall, it appears that *Bbss* N40 and *Bg* SKB40 alter amino acid and energy metabolite concentrations differently than B313 and *Ba* A91. All of the medium samples incubated with the different *Borrelia*-strains differ significantly from the control medium. Interestingly, the overall variability of the metabolites in the control medium samples is higher than the variability in *Borrelia*-culture samples. However, there is no clear explanation for this phenomenon. All sample and data handling steps were identical in the case of every specimen.

The above observations imply that *Bbss* N40 and *Bg* SKB40 have different metabolic rates compared to *Bbss* B313 and *Ba* A91. This finding is rather interesting since it might be expected that the two *Bbss* strains would cause more or less similar metabolic changes. One explanation for the difference between the two *Bbss* strains might be the different plasmid content of the strains, since *Bbss* N40 contains 16 plasmids, while the genetically modified laboratory strain *Bbss* B313 carries only six plasmids. Plasmid contents of *Bg* SKB40 and *Ba* A91 have not been characterized^22–24^.

One weakness of Experiment I is that the concentration fold change differences between the groups are small despite the statistical significance. Therefore, no far reaching conclusions were made based only on these results, thus we continued the study with mouse experiments.

In Experiments II and III, we analyzed both serum and urine samples, because these sample materials are also relevant and easy to collect for human diagnostic purposes. In both experiments, statistically significant differences in the metabolomes of the infected and the control mouse sera and urine samples were detected. In Experiments II and/or III, differences were observed in the serum concentration of L-phenylalanine, L-threonine, L-glutamine, L-leucine, D-glucose, L-lactic acid, citric acid, urea and 2-hydroxybutyric acid (Table 1). The urine metabolite differences were identical in both Experiments II and III. Metabolites with different concentrations in urine samples of the infected and control mice were L-glutamine, L-glycine, acetic acid, allantoin, trigonelline, hippuric acid, indoxyl sulphate and creatinine (Table 2).

The observed metabolites can be divided in four classes: amino acids, energy metabolites, aromatic compounds and others. According to our interpretation, the most interesting findings are the differences in amino acid and aromatic compound concentrations. These differences were mainly observed in the serum, probably because amino acids are found in much smaller concentrations in urine than in serum when the kidney function is normal.

One of the most interesting findings was the increased L-phenylalanine concentration in the infected mice compared with the control ones. Wannemacher *et al*. have shown that serum L-phenylalanine concentrations and phenylalanine-tyrosine ratio significantly increase during bacterial (gram positive and negative) and viral infections^25^ and this appears to be the case also in the *Borrelia* infection. Interestingly, the concentration of L-phenylalanine was significantly increased also in the *Borrelia* culture medium samples. Interesting is also the increased L-threonine concentration in infected mice since L-threonine is known to have immunostimulatory properties, which might explain the increased concentration^26,27^.

One interesting finding in this study was that L-glutamine and urea concentration differences were opposite in serum and urine samples. It is unclear why these metabolites occur in higher concentrations in control mouse sera and, on the other hand, in the urine infected mice. This phenomenon might, however, be due to the different NMR pulse sequences used to analyze the different sample materials. The CPMG sequence, used for the analysis of serum samples, is designed to reduce protein and lipid signals. However, it might also unintentionally reduce signals of small molecules locating close to protein or lipid signals. This phenomenon doesn’t appear with 1D-NOESY used for the analysis of urine samples, thus perhaps explaining the contradictory results^28^.

Another group of metabolites with differing concentrations is formed of the aromatic substances, allantoin (uric acid metabolite), trigonelline (a vitamin B3 metabolite), hippuric acid (metabolite of phenolic compounds) and indoxyl sulphate (L-tryptophan metabolite). All of these are common urine metabolites^29^, which in our study were observed in statistically significantly higher concentrations in the urine samples of control mice. Interestingly, the fold change differences of these metabolites are relatively large, although the role of the differences is difficult to explain. If the clear differences in concentrations of these metabolites appeared also in human samples, they might be potential indicators of human LB but this must be investigated in further studies.

Importantly, the results of our study are mainly in line with the results of earlier studies performed with human LB serum and urine samples^15–17^. Several altered metabolic routes reported in these publications (L-phenylalanine, citric acid cycle and vitamin B metabolism) were also observed in our study. In addition, several other metabolites with changed concentration were found in the present study which were not detected in the previous human studies and the other way around. This can most likely be explained by the different analytical methods used and by the different host species.

Analysis of urinary bladder was included in Experiment III to find out whether NMR is a functional tool to detect *Borrelia* infection in a sample of infected mouse tissue. Previous results of our group have shown that urinary bladder samples are always *Borrelia* culture positive, and therefore, we used this tissue type in our solid-state NMR analysis^30,31^. However, no differences were discovered between infected and control mouse bladders.

In conclusion, the three experiments with three different sample types resulted in a representative picture of *Borrelia* infection NMR metabolome. These results, however, are not a comprehensive representation of the effects of *Borrelia* infection on the metabolite profile of mice due to the obvious lack of sensitivity of the method. There were, however, some identified metabolites, for example L-phenylalanine and allantoin, with biomarker potential, but their applicability for biomarker use remains to be determined. In future studies, it appears to be necessary to use more sensitive methods, such as ultra-high pressure liquid chromatography-mass spectrometry (UHPLC-MS/MS), to be able to identify biomarkers with high specificity for Lyme borreliosis.

## Materials and Methods

### Experimental design

In our study design, the experiments were divided into three parts (Fig. 1). Experiment I was a growth medium metabolomics study in which we compared metabolite profiles of different *Borrelia* strains grown in Barbour-Stoenner-Kelly II (BSK-II) medium. The study was followed by two mouse experiments (Exp. II and III) in which serum and urine sample metabolomes of *Borrelia*-infected and control mice were analyzed by NMR and compared by PCA. Experiment II was a preliminary experiment with 16 mice, and Experiment III was a confirmatory study with 40 mice. In Experiment III, the urinary bladders of the mice were also analyzed using a semi-solid state NMR method.

### Bacteria and bacterial culture

We used four different *Borrelia* strains: *B. burgdorferi* ss N40 (*Bbss* N40), *B. burgdorferi* ss 313 (*Bbss* 313), *B.garinii* SKB40 (*Bg* SKB40) and *B. afzelii* A91 (*Ba* A91). All of these strains were used in the growth medium experiment, while the *in vivo* experiments were performed using only *Bbss* N40.

Bacteria were grown in BSK II medium at 33 °C. Bacteria used for the infection were at the stationary growth phase. Before infection, the bacteria were washed with PBS, diluted to the 10^7^ bacteria/ml concentration in PBS and then transferred to a syringe for mouse infection.

To verify the infection status of the mice, ear skin samples were collected in Experiments II and III at the end of the study. Collected skin samples were placed in BSK II medium at 33 °C for *Borrelia* cultivation. Rifampicin (5 µg/ml) and phosphomycin (10 µg/ml) were added to the culture medium to avoid contaminating bacterial growth. The cultures were observed every two weeks for six weeks by dark field microscopy.

### Experiment I: *In vitro* NMR metabolome of borrelia

Four different *Borrelia* strains (*Bbss* N40, *Bbss* 313, *Bg* SKB40, *Ba* A91) were cultured in BSK-II medium at 33 °C as six parallel samples for four days until the bacteria reached a stationary growth phase. The cultures were then centrifuged at 3000 rpm for five minutes to remove the bacterial cells. Supernatant was further sterilized by passing the medium through 0.22 µm filters. As a control, two samples of BSK-II medium without bacteria were incubated and processed similarly. In total, the number of samples was 30 including six control medium samples and six medium samples after cultivation of each of the four strains. After incubation and processing, sample aliquots of 600 µl were stored at −80 °C.

### Experiments II and III: *In vivo* metabolome of borrelia infection in mice

In the preliminary mouse Experiment II, 16 female C3H/HeNHsd mice (Envigo, Netherlands), and in the confirmatory Experiment III, 40 mice, were used as *in vivo* models of *Borrelia* infection. In both experiments, half of the mice were *Bbss* N40-infected, while the other half formed the control group receiving PBS injections. Mice were infected by intradermal injection, containing 10^6^ bacteria in 100 µl of PBS, in the lower back. Infected and control mice were placed in different cages, four mice/cage, but otherwise treated equally. Water and food were given *ad libitum* during the whole study. Mice were observed weekly to monitor their general health. Ear markings were used for mouse identification.

Urine samples were collected on the fouth week after infection on three consecutive days before the endpoint of the experiment. Urine samples were collected by placing mice on a plastic-covered paper, where the mice were allowed to urinate freely. Urine drops were collected from the paper by pipetting to sterile Eppendorf tubes. The samples of each animal from all collection time points were pooled together.

On the week 4 after infection, the mice were sacrificed by cardiac puncture under isoflurane anesthesia followed by neck break. The total blood sample from each mouse was collected by cardiac puncture with a heparinized syringe and placed in 1.5 ml Eppendorf tubes on ice. If cardiac puncture resulted in blood volume under 0.8 ml, the mouse’s chest was opened and puncture performed directly to the heart. This procedure was successful in most cases, but resulted in hemolysis disturbing the NMR analysis of the serum samples. After mice were sacrificed, ear skin samples were collected for *Borrelia* culture. Urinary bladders were collected for semi-solid state NMR in Experiment III. Forceps and scissors used for sample collection were disinfected with 70% ethanol and rinsed with sterile water after every mouse during tissue sample collection.

After sample collection, all urine samples were placed in Eppendorf tubes at −80 °C. Blood samples were first centrifuged at 1000 g for 10 min and serum was carefully pipetted avoiding erythrocyte contamination. Serum was transferred to a new tube and placed also in −80 °C. Urinary bladders for semi-solid state NMR were weighted and placed in the semi-solid state NMR adapters. Approximately 10 µl of deuterated phosphate buffer (0.1 M KH_2_PO_4_ + 2 mM NaN_3_ + 0.1% 3-(trimethylsilyl)propionic-2,2,3,3-d4 acid sodium salt (TSP)) was added, and the adapter was closed before freezing it with liquid nitrogen. Samples were stored in −80 °C until NMR measurements.

Animal experiments in this project were conducted according to the recommendations of the Finnish Act of the Use of Animals for experimental purposes of the ministry of agriculture and forestry of Finland and the principle of 3 R. All efforts were made to minimize the suffering of the animals. All experimental infection studies were approved by the National Animal Experiment Board in Finland (permission ESAVI/6265/04.10.07/2017).

### Sample handling/processing for NMR

Medium samples of Experiment I were first freeze-dried for 48 h to remove water. Before the NMR analyses, the samples were dissolved in 600 µl of buffer solution (0.5 M phosphate buffer, pH 7.0, 0.1 m-% TSP, 2 mM NaN3, pH adjusted with NaOD and DCl (D meaning deuterium, ^2^H isotope of hydrogen)) by vortexing for 15 minutes. Then, the samples (600 µl) were transferred into 5 mm NMR tubes (Cambridge Isotope Laboratories, Inc., Andover, MA).

Serum samples of Experiments II and III were thawed on ice and thoroughly mixed in a shaker. 200 µl serum was mixed with 400 µl 1 M NaCl in D_2_O, samples were centrifuged 1650 g 5 min 4 °C and 540 µl sample was mixed with 60 µl deuterated 1.5 M KH_2_PO_4_ + 2 mM NaN_3_ + 0.1% TSP buffer. Samples were transferred to 5 mm NMR tubes (VWR, Randor, Pennsylvania, USA) and stored at 4 °C until measurements. Sodium azide was used in the sample buffer to prevent bacterial growth during storage.

Urine samples of Experiments II and III were thawed on ice, thoroughly mixed in a shaker, centrifuged 1650 g 7.5 min +4 °C, and 120 µl of the supernatant was mixed with 60 µl 1.5 M KH_2_PO_4_ + 2 mM NaN_3_ + 0.1% TSP buffer. Urine samples were transferred to 3 mm NMR tubes (VWR) and stored at +4 °C until measurements^32^. Due to the low amount of urine samples, the samples of one animal from all collection time points were pooled.

### NMR experiments

NMR measurements of growth medium samples were performed using a Bruker Avance 500 MHz spectrometer with BBI room temperature probe (Bruker BioSpin AG, Fällanden, Switzerland) operating at 500.13 MHz for ^1^H at 25 °C in batches of five samples in random order.

NMR experiments on serum and urine samples were conducted by Bruker Avance III 600 MHz instrument with TCI PRODIGY cryoprobe (Bruker BioSpin AG, Fällanden, Switzerland) operating at 600.16 MHz for ^1^H. SampleJet automatic sample changer (Bruker BioSpin AG, Fällanden, Switzerland) was utilized to store samples at 4 °C before and between measurements, and to change samples automatically. All samples were pre-warmed for 10 minutes before measurement in order to do the measurements at 25 °C. Sample measurement order was randomized.

NMR experiments on urinary bladders were conducted by Bruker Avance III 400 MHz (Bruker BioSpin AG, Fällanden, Switzerland) instrument with HR-MAS 4 mm solid state probe. The spinning rate was 4000 Hz and the samples were measured at 4 °C to reduce tissue degradation and metabolic changes. For the measurement, the samples were first placed in 4 mm ZrO_2_ rotors inside the inserts directly from the freezer, and the rotor was then placed inside the magnet. Shimming and tuning were done manually for each sample. The measurement was started within 20 minutes after taking the sample out of the freezer. Sample measurement order was randomized.

Growth medium NMR measurements were performed using a Bruker Avance 500 MHz spectrometer (Bruker BioSpin AG, Fällanden, Switzerland) operating at 500.13 MHz for ^1^H at 25 °C in batches of five samples in random order and CPMG, 1D-NOESY, DQF-COSY (double-quantum filtered correlation spectroscopy), CH_2_-modified HSQC (heteronuclear single quantum coherence), NOESY and TOCSY (total correlated spectroscopy) pulse sequences were utilized. From serum and urinary bladder samples, CPMG spectra were measured. CPMG pulse sequence with presaturation was used in order to reduce the water signal and broad signals caused by lipids and proteins which cover small molecule signals. Urine samples, naturally containing only low concentrations of proteins and lipids, were measured with 1D-NOESY pulse sequence with presaturation to reduce only the water signal.

The following parameters were used in the CPMG measurement of serum samples: acquisition time 3.41 s, echo time (d20) 0.0015 s, loop counter (l4) 64, 90° pulse length (p1) 10.65 µs and relaxation delay (d1) 5.0 s. Spectral width was 16 ppm, 64 k data points were collected, and number of scans was 256.

All 1D-NOESY measurements of urine samples were also conducted using the following parameters: acquisition time 3.41 s, mixing time (d8) 0.10 s, p1 9.0 µs and d1 5.0 s. Spectral width was 16 ppm and 64 k data points were collected, number of scans was 128.

The following parameters were used in the CPMG measurement of urine bladder samples: acquisition time 2.56 s, d20 0.0015 s, l4 128 and d1 5.0 s. Spectral width was 16 ppm, 32 k data points were collected and number of scans was 256. P1 was determined for each sample, and ranged from 4.45 to 5.80 µs.

Growth medium sample analyses were conducted with the following parameters: The pulse program used was CPMGPR with water suppression (4.71 ppm) and exclusion of the large biomolecules using the T2 filter. Acquisition time for one scan of 1.64 µs, a 90° pulse of 6.90 µs and four dummy scans were used. The total FID was a sum of 256 scans collected with 64 k data points. The dwell time between two data points in the FID was 50 µs in spectral width of 20 Hz, and line broadening was set to 0.30 Hz. TSP was used as an internal reference compound.

### NMR data processing

NMR data was handled with Bruker TopSpin 3.5 pl3 software (Bruker BioSpin GmbH, Rheinstetten, Germany). All spectra were manually phase corrected, baseline corrected using polynomic function method and calibrated using α-glucose or TSP as reference signal. Data was normalized to total spectral area.

After manual spectra correction, data were bucketed for statistical analyses by Bruker Amix 3.9.15 software (Bruker BioSpin GmbH, Rheinstetten, Germany). Bucket size 0.01 ppm was used for all other spectra, but growth medium spectra were bucketed in 0.05 ppm buckets. A larger bucket size was used in growth medium dataset in order to minimize the effect of pH and varying ion content induced signal position variation, which were observed in the medium samples. Data was normalized to the whole spectrum integral in a manner that the whole spectrum integral is assumed to be 1. Bucket tables were transferred to Microsoft Excel 2013, where data was transformed from text to numeric format. Peak alignment correction of urine data was conducted by matlab based *i*coshift algorithm^33^.

All of the results presented in this study are based on a three step data analysis approach. First, PCA analysis was done to obtain unsupervised information of metabolite differences between sample types. Secondly, PLS-DA was used as a supervised model to confirm the differences observed in PCA. Thirdly, statistical significances of differences observed in PCA and PLS-DA were determined by univariate statistics.

Numeric data were analyzed using multivariate analysis by Umetrics Simca-P +12.0.1 software (Umetrics, Umeå, Sweden). In both urine and serum data analyses, Pareto scaling of data was used and analysis itself was conducted using PCA modeling (unsupervised linear mixture model which explains the variance within a dataset) as the main analysis, and PLS-DA (Partial least square discriminant analysis) as a confirmatory analysis.

In our dataset, the principal components are vectors of metabolite contributions. All principal components are decorrelated to each other. Unsupervised PCA is used as linear mixture model, in which the data matrix is divided into two parts. One is a score matrix, which contains the positions of the observations, and the other is a loading matrix, which contains the weights for the original variables. Supervised PLS-DA relates the multivariate metabolomic data to the response vector by a linear regression model and attempts to find an optimal decomposition of the predictor dataset^34^.

The scaling for PCA and PLS-DA was selected in order to achieve the highest possible *R*^2^ and *Q*^2^ values. *R*^2^ represents the variation of the model, whereas *Q*^2^ is an estimate of the predictive ability of the model^35,36^. One (1) is the highest value of both parameters^35^. *R*^2^ is over 0.8 when the model is considered to be reliable, and the difference between *R*^2^ and *Q*^2^ should not exceed 0.2.

The presentation of results was made using a scores plots to show differences between samples and loadings plots to show which metabolites differentiate in concentration between the samples of infected and control mice.

Based on the multivariate results, univariate analysis was performed to determine the statistical significance of the concentration differences of each metabolite observed in PCA and in PLS-DA. Univariate analysis was performed using merged bucket values corresponding to the area of analyzed metabolite’s ^1^H signal. The data was normalized to the total spectral area. Statistical analyses were performed using JMP Pro 13.1.0 (SAS, Cary, NC, USA) software. The normal distribution of the data was first tested using Shapiro-Wilk W –test. If the relative integral values of a metabolite were normally distributed, Student’s t-test was used in Experiment I and t-test was used in Experiments II and III to analyze statistical significance. If the data was not normally distributed, Kruskal-Wallis test (Exp. I) or Mann-Whitney U test (Exp. II and III) was used. The difference was regarded as statistically significant if p-value was <0.05.

Signal identification was performed using the Human Metabolome Database (HMDB)^37–39^ and literature searches^29,40^. Raw identification of the metabolites was made based on the literature, followed by confirmation using HMDB.

## Supplementary information

Supplementary information

## Data Availability

The data of this project will be deposited to IDA to ensure that the research community has a long-term access to the data (https://openscience.fi/ida). The service is offered to Finnish universities and polytechnics by the Ministry of Education and Culture in Finland. All metadata of Metabo-Lyme project are made available in Etsin (https://etsin.avointiede.fi/). Etsin offers access to datasets in various fields via a joint metadata model. The descriptive metadata stored in the service includes information on the authors, subject, format and licensing of the dataset. Anyone can use Etsin to search for research datasets.
