# Supplementary material for: NMR metabolome of Borrelia burgdorferi in vitro and in vivo in mice
Source: Sci Rep. 2019 May 29;9:8049. doi: 10.1038/s41598-019-44540-5 (PMC6541645; doi:10.1038/s41598-019-44540-5)
Supplement: Supplementary file 1 — Supplementary information [file 41598_2019_44540_MOESM1_ESM.docx]

NMR metabolome of *Borrelia burgdorferi in vitro* and *in vivo* in mice

Authors: Otto Glader^1*^, Elina Puljula^2,3^, Johanna Jokioja^2^, Maarit Karonen^3^, Jari Sinkkonen^2^, Jukka Hytönen^1,4^

Affiliations:

^1^ Institute of Biomedicine, University of Turku, Turku, Finland.

^2^ Instrument Centre, Department of Chemistry, University of Turku, Turku, Finland.

^3^ Natural Compound Chemistry, Department of Chemistry, University of Turku, Turku, Finland.

^4^ Clinical Microbiology, Turku University Hospital, Turku, Finland

Corresponding author: Otto Glader, otto.o.glader@utu.fi

Table S1. The compounds identified in growth medium samples (lyophilized, in phosphate buffer in D_2_O, pH 7.0, 25 °C) after cultivating *Bbss* N40 using ^1^H 500 MHz NMR. Metabolites are presented from smaller to larger ^1^H signal ppm value. Metabolites that differ in concentration between bacterial strains and control medium are indicated by bold.

| Compound | Position | Multiplicity *^a^* | δ_H_ (ppm) | *J* (Hz) |
| --- | --- | --- | --- | --- |
| **Isoleucine** | *δ*-C**H_3_** | t | 0.96 | 7.4 |
|  | *β*-C**H_3_** | d | 1.03 | 7.7 |
|  | *α*-C**H** | d | 3.66 | 4.3 |
| **Leucine** | *δ*-C**H_3_,** *δ´*-C**H_3_** | t | 0.98 | 5.5 |
| **Valine** | *γ*-C**H_3_** | d | 1.01 | 7.0 |
|  | *γ´*-C**H_3_** | d | 1.06 | 7.3 |
|  | *β*-C**H** | m | 2.22–2.34 | - |
| **Threonine** | *γ*-C**H_3_** | d | 1.35^b^ | 6.6 |
|  | *α*-C**H** | d | 3.62 | 4.9 |
|  | *γ*-**H** | m | 4.25–4.31 | - |
| **Lactic acid** | **H**3 | d | 1.35 | 7.0 |
|  | **H**2 | q | 4.14 | 7.0 |
| **Alanine** | *β*-C**H_3_** | d | 1.50 | 7.3 |
| Lysine | *δ*-C**H_2_** | m | 1.69–1.79 | - |
|  | *ε*-C**H_2_** | t | 3.04 | 7.5 |
| Acetic acid | -C**H_3_** | s | 1.94 | - |
| Proline | *γ*-C**H_2_** | m | 1.95–2.06 | - |
|  | *δ*-C**H_2_** | m | 3.33–3.39 | - |
| **β*-N*-Acetyl-d-glucosamine** | -NH-CO-C**H_3_** | s | 2.08 | - |
|  | **H**1 | d | 4.76 | 8.4 |
| α*-N*-Acetyl-d-glucosamine | -NH-CO-C**H_3_** | s | 2.08 | - |
|  | **H**1 | d | 5.23 | 3.5 |
| **Methionine** | *δ*-C**H_3_** | m | 2.16–2.26 | - |
|  | *γ*-C**H_2_** | t | 2.66 | 7.8 |
| Pyruvic acid | **H**3 | s | 2.39 | - |
| Succinic acid | **H**2, **H**2`, **H**3, **H**3` | s | 2.42 | - |
| **Citric acid** | **H**3a, H3a´ | m | 2.51-2.54 | - |
|  | **H**3b, H3b´ | m | 2.64-2.67 | - |
| Aspartic acid | *β*-C**H_2_** | dd | 2.73 | 8.0; 17.4 |
|  | *β*-C**H_2_** | dd | 2.83 | 4.0; 17.4 |
| Choline | -C**H_3_** | s | 3.18 | - |
| Glycine | *α*-C**H_2_** | s | 3.59 | - |
| **β-d-Glucose** | **H**1 | d | 4.68 | 8.0 |
| **α-d-Glucose** | **H**1 | d | 5.26 | 3.8 |
| **Tyrosine** | *δ*-C**H,** *δ`*-C**H** | d | 6.93 | 8.6 |
|  | *ε*-C**H,** *ε`*-C**H** | d | 7.21 | 8.6 |
| **Phenylalanine** | *δ*-C**H,** *δ`*-C**H** | d | 7.35 | 7.0 |
|  | *ζ*-C**H** | m | 7.37–7.41 | - |
|  | *ε*-C**H,** *ε`*-C**H** | m | 7.41–7.48 | - |
| Formic acid | C**H** | s | 8.48 | - |

*^a^* Multiplicity indicates how many neighbouring hydrogen nuclei the signal consists of. (s, singlet; d, duplet; dd, duplet of duplets; t, triplet; q, quartet; m, multiplet)
*^b^* Detected in the control samples, the signal is overlapping with the duplet of lactic acid.


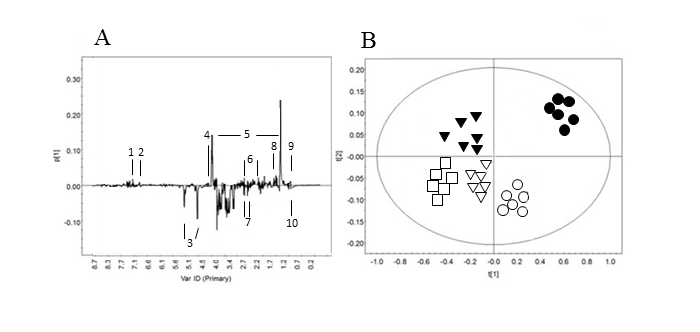


*R*^2^ 0.98
*Q*^2^ 0.97


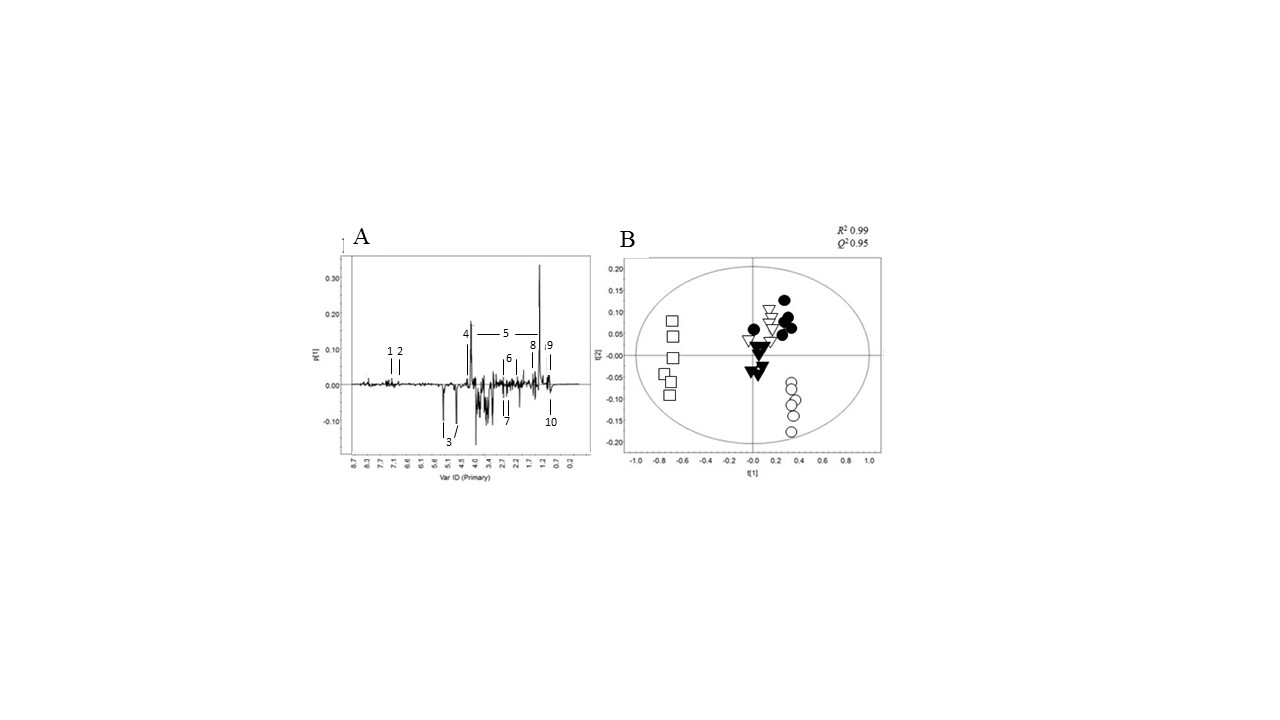


*R*^2^ 0.99
*Q*^2^ 0.95

C

D

Figure S1. *In vitro* metabolome of different *Borrelia* strains. In the loadings plot panels (PCA: A and PLS-DA: C) the metabolome differences between control and *Bbss* N40 growth media are shown. Identified metabolites in the loadings plots: 1: L-phenylalanine, 2: L-tyrosine, 3: D-glucose, 4: L-threonine, 5: L-lactic acid, 6: L-methionine, 7: citric acid, 8: L-alanine, 9: L-valine, 10: L-leucine, L-isoleucine. Positive y-axis represents *Borrelia* growth medium samples and negative y-axis represents control medium samples in both plots. Same annotation are used in both plots to highlight the small differences between PCA and PLS-DA results.

The scores plot panels (PCA: B and PLS-DA: D) of growth media show that all culture medium samples (n = 30) differ considerably from each other forming own patterns after growth of different *Borrelia* strains. Empty squares represent control medium, empty triangles *Bbss* N40, black triangles *Bbss* 313, black circles *Bg* SKB40 and empty circles *Ba* A91.

##
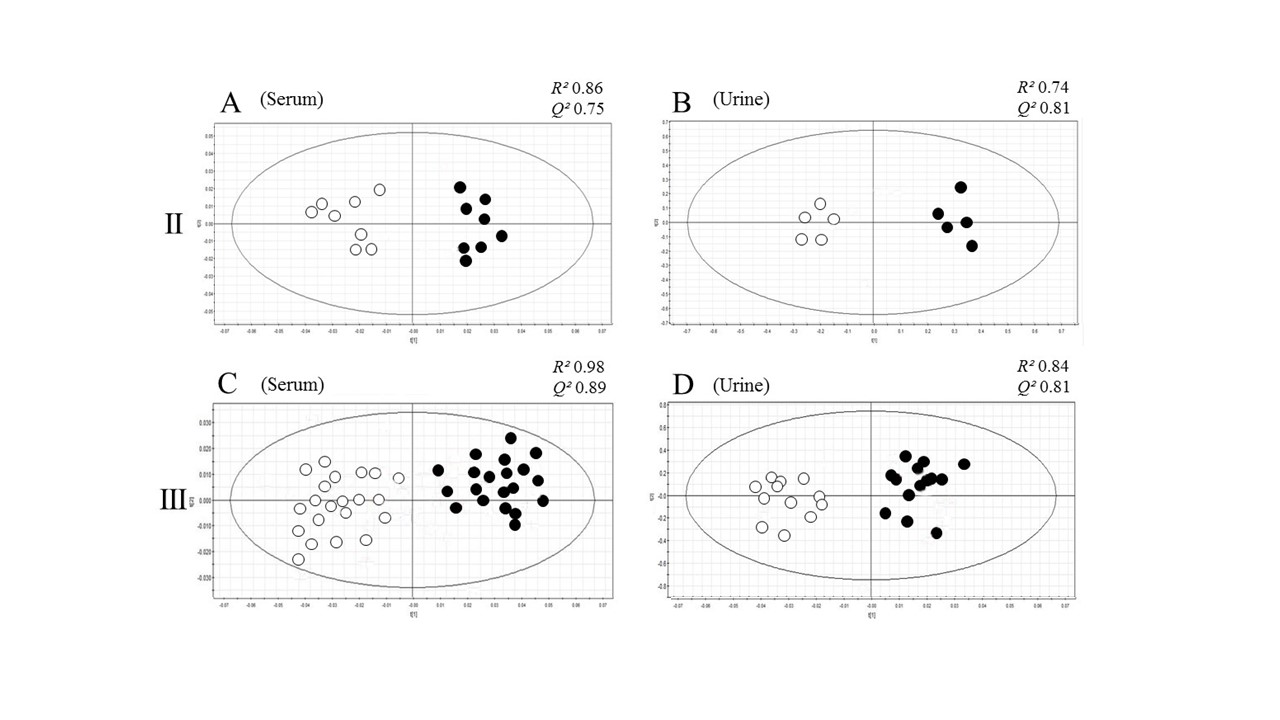
Figure S2. Metabolome differences of *Borrelia* infected and control mice. Scores plot figures of multivariate Partial least squares discriminant analysis (PLS-DA) of Experiment II and III serum and urine samples (A: Exp. II serum, B: Exp. II urine, C: Exp. III serum, D: Exp. III urine) show the difference between infected mouse (black circles) and control mouse metabolomes (empty circles). *R^2^* and *Q^2^* values, representing the reliability of the model, are shown above each scatter plot. The values indicate that the results of all analyses, except the PLS-DA of urine in Experiment II, are reliable.


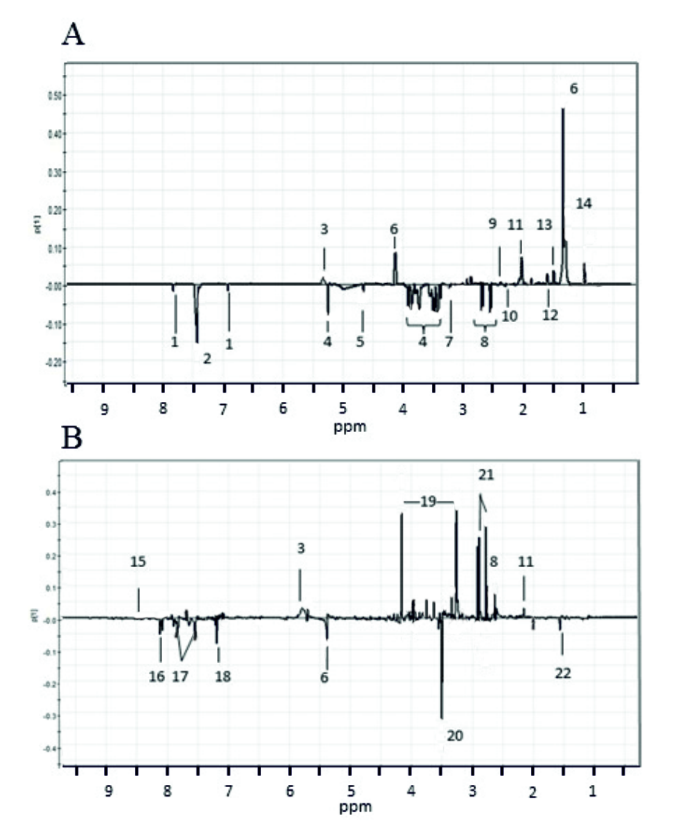


Figure S3. Loadings plots of the serum (A) and urine (B) analyses of Experiment III show the differentiating metabolites in PLS-DA analysis. Positive y-axis represents metabolites occurring in higher concentrations in the control mice, and negative y-axis represents metabolites occurring in higher concentrations in the infected mice. Same annotations are used in this figure as in Figure 4, in order to highlight the small differences between analyses. Identified metabolites are: 1: L-histidine, 2: L-phenylalanine, 3: urea, 4: D-glucose, 5: L-threonine, 6: L-lactic acid, 7: carnitine, 8: citric acid, 9: pyruvic acid, 10: L-valine, 11: L-glutamine, 12: 2-hydroxybutyric acid, 13: L-alanine, 14: L-leucine, 15: formic acid, 16: trigonelline, 17: hippuric acid, 18: indoxyl sulphate, 19: allantoin, 20: creatinine, 21: L-glycine, 22: acetic acid.


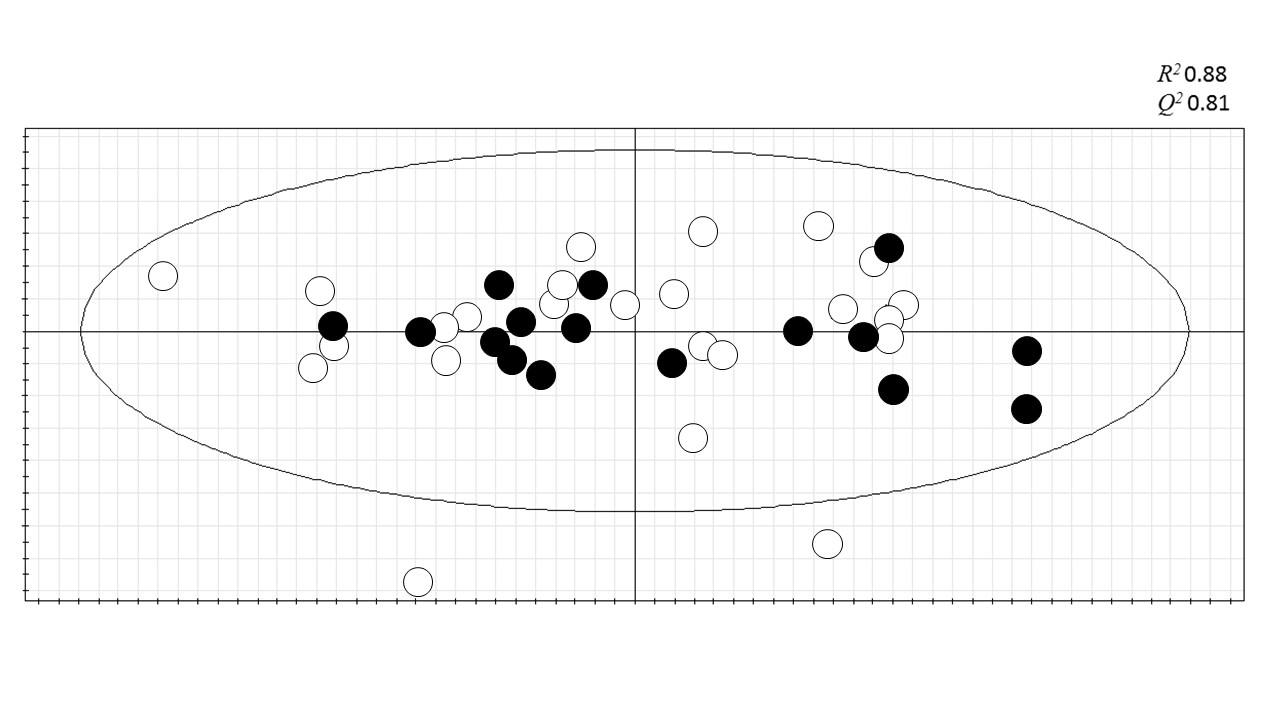


Fig S4. Scatter blot figure of PCA of the Experiment III urinary bladder samples. Infected mouse samples are shown with black circles and control mice samples with empty circles. No difference is observed between infected and control samples.
